# A synthetic glycodendropeptide induces methylation changes on regulatory T cells linked to tolerant responses in anaphylactic-mice

**DOI:** 10.3389/fimmu.2023.1165852

**Published:** 2023-06-02

**Authors:** Rafael Núñez, María J. Rodríguez, Clara Lebrón-Martín, María del Carmen Martín-Astorga, Javier Ramos-Soriano, Javier Rojo, María J. Torres, José A. Cañas, Cristobalina Mayorga

**Affiliations:** ^1^ Laboratory of Allergy, Allergy Research Group, Instituto de Investigación Biomédica de Málaga-Plataforma BIONAND (IBIMA-BIONAND), Málaga, Spain; ^2^ Department of Medicine, Universidad de Málaga (UMA), Málaga, Spain; ^3^ Laboratory of Glycosystems, Institute of Chemical Research (IIQ), Centro Superior de Investigaciones Científicas (CSIC) - Universidad de Sevilla, Sevilla, Spain; ^4^ Clinical Unit of Allergy, Hospital Regional Universitario de Málaga, Málaga, Spain

**Keywords:** regulatory T cells, methylation changes, immunotherapy, tolerance, lipid transfer proteins, food allergy, glycodendropeptide

## Abstract

**Introduction:**

Lipid transfer proteins (LTPs) are allergens found in a wide range of plant-foods. Specifically, Pru p 3, the major allergen of peach, is commonly responsible for severe allergic reactions. The need for new alternatives to conventional food allergy treatments, like restrictive diets, suggests allergen immunotherapy as a promising option. It has been demonstrated that sublingual immunotherapy (SLIT) with synthetic glycodendropeptides, such as D1ManPrup3, containing mannose and Pru p 3 peptides induced tolerance in mice and that the persistence of this effect depends on treatment dose (2nM or 5nM). Moreover, it produces changes associated with differential gene expression and methylation profile of dendritic cells, as well as phenotypical changes in regulatory T cells (Treg). However, there are no works addressing the study of epigenetic changes in terms of methylation in the cell subsets that sustain tolerant responses, Treg. Therefore, in this work, DNA methylation changes in splenic-Treg from Pru p 3 anaphylactic mice were evaluated.

**Methods:**

It was performed by whole genome bisulphite sequencing comparing SLIT-D1ManPrup3 treated mice: tolerant (2nM D1ManPrup3), desensitized (5nM D1ManPrup3), and sensitized but not treated (antigen-only), with anaphylactic mice.

**Results:**

Most of the methylation changes were found in the gene promoters from both SLIT-treated groups, desensitized (1,580) and tolerant (1,576), followed by the antigen-only (1,151) group. Although tolerant and desensitized mice showed a similar number of methylation changes, only 445 genes were shared in both. Remarkably, interesting methylation changes were observed on the promoter regions of critical transcription factors for Treg function like *Stat4*, *Stat5a*, *Stat5b*, *Foxp3*, and *Gata3*. In fact, *Foxp3* was observed exclusively as hypomethylated in tolerant group, whereas *Gata3* was only hypomethylated in the desensitized mice.

**Discussion:**

In conclusion, diverse D1ManPrup3 doses induce different responses (tolerance or desensitization) in mice, which are reflected by differential methylation changes in Tregs.

## 1 Introduction

Over last years, food allergy (FA) has increased worldwide in both adult and childhood populations, reaching a prevalence around 6% in certain regions of Europe (1). It constitutes one of the most widespread diseases nowadays and represents a serious health and economic burden (2). FA is an adverse immune reaction to food proteins, usually IgE-mediated (3). Moreover, this is a heterogeneous condition because of the diversity of factors that influence it: patient’s genetics, lifestyle, or the type of food allergen (4).

One of the most common food allergens in adults is non-specific lipid-transfer proteins (nsLTPs), especially Pru p 3, which is the major allergen of peach. LTPs are widespread in multiples foods, although they are more relevant in plant-foods, where they display a defensive function (5). Their structure is very stable and conserved, and resists degradation by heat or extreme environments factors such as gastric acids (6). The main problem is that, due to their conserved structure, they can produce cross-reactivity with nsLTP from other foods, inducing severe reactions. Thus, these characteristics of nsLTPs cause a complex clinical pattern known as LTP syndrome (7).

Regarding management and therapy, in most cases avoiding the food(s) containing these allergens is the only solution. Nevertheless, it worsens the quality of life of patients and an involuntary intake of allergen produces a high risk of suffering a serious allergic reaction, which also increases the patient’s fear and anxiety (8). Therefore, it is necessary to develop a safe and effective treatment offering allergic patients a permanent improvement. Allergen specific immunotherapy (AIT) represents the best option in these cases (9). In this context, our group has shown that sublingual immunotherapy (SLIT) with Pru p 3 in peach- and peanut-allergic patients can offer great results, increasing the quantity of food that they can tolerate and decreasing allergic reactions (10). Moreover, regarding cellular and immunological mechanisms, SLIT with Pru p 3 induces the production of Pru p 3 specific IgG_4_ with anti-inflammatory properties, and the change of the pro-inflammatory phenotype of dendritic cells towards a tolerogenic profile by increasing molecules as programmed cell death ligand-1 (PD-L1 or CD274) (11).

However, AIT using whole natural extract or specific allergen has several drawbacks, such as high-variability, dosing-problem, and lack of security in patients with some FA (12). Therefore, there is a need for developing other immunotherapy approaches to overcome these flaws. A good alternative could be the use of synthetic glycodendropeptides with the epitope of the allergen (13). Our research group has developed a synthetic nanosystem based on a dendritic scaffold containing 9 mannoses and a T cell Pru p 3 epitope (13), called D1ManPrup3, demonstrating their capacity to induce tolerance (at 2 nM) or desensitization (at 5 nM) in an anaphylactic mouse model depending on dosage (14). In this study, a prevention of anaphylaxis was observed after D1ManPrup3-SLIT (with neither a decrease in body temperature nor severe behavioral symptoms), as well as a decrease in Cd4^+^ proliferative responses. Interestingly, mice treated with D1ManPrup3 showed a higher percentage of Cd4^+^/Cd25^+^/Foxp3^+^ cells, defined as regulatory T cells (Treg) (14), which play a crucial role in the maintenance of tolerant responses.

Treg is described as a population of Cd4^+^ T cells that expresses the antigen Cd25 (α-chain of IL-2 receptor), which can promote regulatory responses to mitigate immune and allergic diseases. Different subpopulations of Treg have been established, but the most important ones are those that express the forkhead box protein 3 (Foxp3) transcription factor, which has been considered a Treg cell maker essential to develop their functions (15). Moreover, there is evidence of Treg providing food tolerance in mice (14), although the specific contribution of Tregs to allergy and oral tolerance in humans remains poorly understood. A recent study in FA demonstrated that oral immunotherapy promoted a delayed and sustained IL-2-dependent activation of Tregs (16).

Besides, the marked, worldwide increase in the cases of FA worldwide during the last decades could indicate that environmental factors influence the genome and promote these allergic diseases at the expense of tolerance (17). Therefore, there has been an increase in the immunological and genetic data demonstrating the central role of Treg in the promotion of allergen tolerance in FA and other inflammatory pathologies (18, 19). However, a proallergic environment can influence the regulatory response of Treg, perpetuating allergic disorders (20). Taking all these data into account, it is necessary to study more in detail the role of Treg in tolerance acquisition in FA and search for long-term biomarkers of an effective immunotherapy.

Epigenetic regulation and, particularly, DNA methylation have been associated with the pathogenesis of FA (21). Similarly, changes in DNA methylation of certain cell types have been related to immunotherapy effects (4). In fact, our research group has recently published a work showing in dendritic cells from an anaphylactic mouse model treated with D1ManPrup3-SLIT differential methylation changes in CpG context (22). Most differentially methylated regions were found on the area of influence of gene promoters linked to immune mechanisms and tolerance responses, such as *Il1a*, *Il1b*, *Il12b*, *Ifng*, and *Tnf*. Moreover, these epigenetic changes could be considered as potential long-term biomarkers of tolerance and effectiveness of immunotherapy, since they last over time and would not be transient (23), which could confer long-term protection to allergic patients.

Therefore, our main objective was to identify the epigenetic changes induced by the immunotherapy through the study of DNA methylation changes in Treg, using Whole Genome Bisulphite sequencing (WGBS-seq). For this, the anaphylactic mice, which was used for developing the different comparison analysis, was considered as basal group. Differences found between desensitized and tolerant animals can be used to obtain prognostic biomarkers of long-term tolerance.

## 2 Materials and methods

### 2.1 Anaphylactic animal model

To develop the anaphylactic mouse model, female Balb/c mice of 4-5 weeks of age (Janvier Lab, Le Genest-Saint-Isle, France) were required. International standards of animal welfare were applied in experimental animal procedures. The Animal Experimentation Ethics Committee of Andalusian Centre for Nanomedicine and Biotechnology (BIONAND, Malaga, Spain) approved all protocols related to animal manipulation.

Mice were separated into four groups: 1) sensitized, non-anaphylactic (antigen-only) (*n* = 7); 2) anaphylactic, non-treated (anaphylactic) (*n* = 7); 3) anaphylactic, treated with 2 nM D1ManPrup3 (tolerant) (*n* = 7); 4) anaphylactic, treated with 5 nM D1ManPrup3 (desensitized) (*n* = 7), according to previously described experimental procedures (22). Briefly, intranasal sensitization was performed once a week for five weeks with 20 µg of natural Pru p 3 (Bial Laboratory, Zamudio, Spain) together with 20 ng of lipopolysaccharide (LPS) (InvivoGen, San Diego, CA, USA) (24), except for antigen-only group which was only sensitized with Pru p 3. After sensitization, SLIT was developed according to previous experimental design (14, 22). Briefly, SLIT was administered once a week for 8 weeks as follows: antigen-only and anaphylactic mice were treated with 1X PBS, and tolerant and desensitized mice with 2 nM or 5nM D1ManPrup3, respectively. Preparation of synthetic glycodendropeptide D1ManPrup3 was performed according to previous protocols, purified by liquid chromatography (HPLC), and characterized by mass spectrometry (13).

One week after the last dose of SLIT, mice were intraperitoneally challenged with 100 µg of natural Pru p 3 (Bial Laboratory), and rectal temperature and behavioral symptoms were evaluated 30-40 min after challenge, according to a scoring system (25). It is at this time when spleens were collected to perform the analysis and experiments. Additionally, five weeks after the last dose of SLIT, intraperitoneal dose of natural Pru p 3 (100 µg) was administered to confirm tolerance or desensitization. Then, mice were bled from retro-orbital plexus, while they were anesthetized, to obtain serum and storage it to -20°C for further humoral analyses. After this procedure, mice were sacrificed by cervical dislocation performed by authorized and trained personnel. Finally, spleen was aseptically collected and disaggregated through a cell strainer 70 µm (Corning, NY, USA) to obtain a single-cell suspension.

### 2.2 Humoral analysis

Pru p 3-specifc serum antibody levels (sIgE and sIgG_1_) were evaluated after Pru p 3-challenge by ELISA using Pru p 3 5 µg/mL for coating 96 wells plates (Corning, Corning, NY). Then, sera were added and Pru p 3-specifc antibodies were revealed using biotinylated labelled rat anti-mouse IgE or IgG_1_ (BD Biosciences Pharmingen, San Diego, CA). ELISA results were expressed as 450nm optical density.

### 2.3 Regulatory T cells isolation

**Figure 1:**
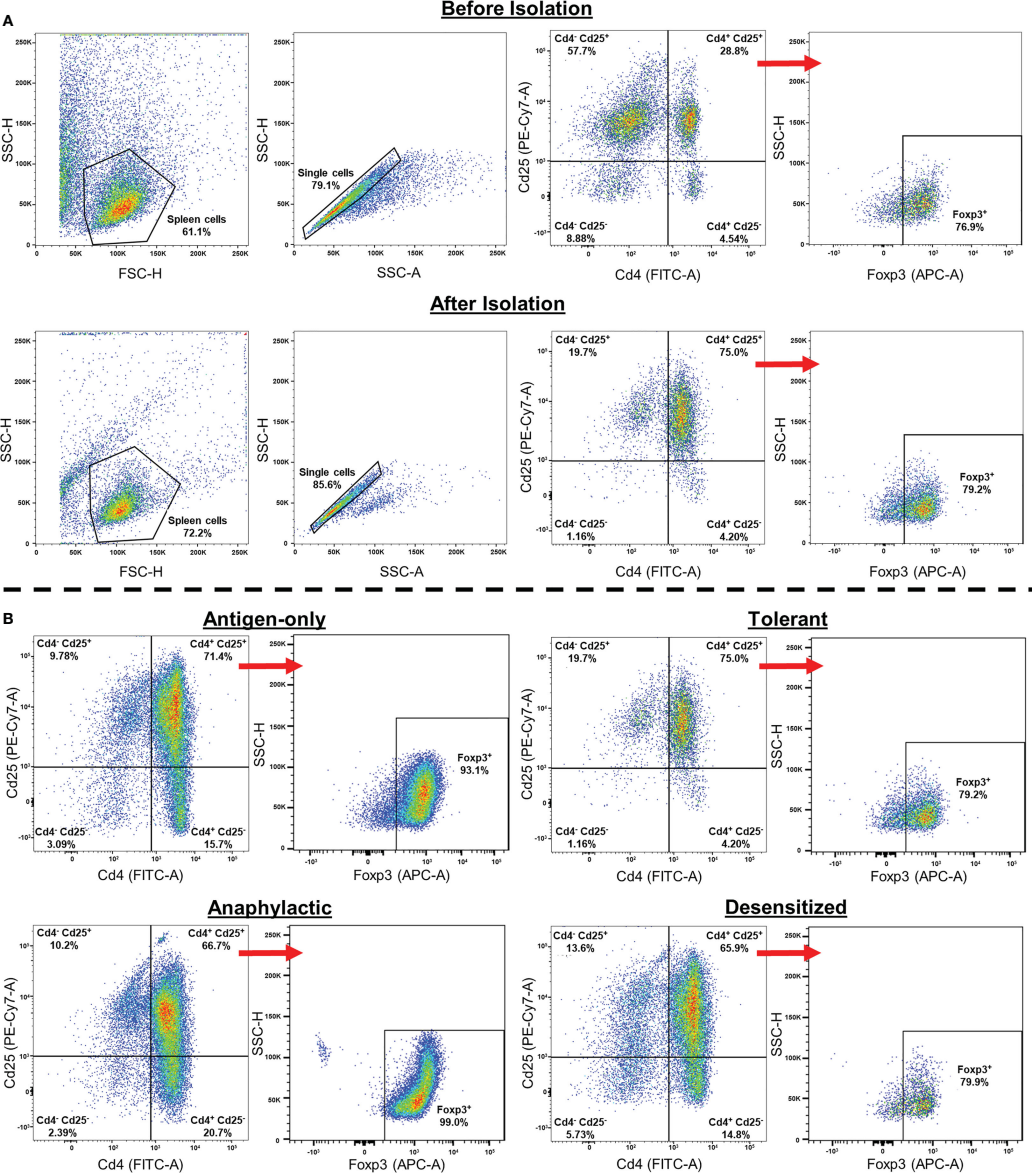
Gate selection strategy used in Cd4^+^/Cd25^+^ isolation. **(A)** Gating strategy followed to confirm the purity of Cd4^+^/Cd25^+^/Foxp3^+^ obtained after Treg isolation. The percentage of the different cell populations before isolation is also shown. **(B)** Representative plots of isolated Cd4^+^/Cd25^+^/Foxp3^+^ cells in the different groups of mice. After isolation the purity of Cd4^+^/Cd25^+^ cells between groups was 65-75%, which contains a high percentage of Foxp3^+^ cells (80-99%).

Treg from spleen were purified by positive magnetic immunoselection using CD4^+^CD25^+^ Regulatory T Cell Isolation Kit (Miltenyi Biotec, Bergisch Gladbach, Germany), following manufacturer’s protocol. **Figure 1** shows flow gating strategy, and the percentage of Cd4^+^/Cd25^+^ population before and after isolation, as well as the proportion of obtained Foxp3^+^. Moreover, the percentage of Cd4^+^/Cd25^+^/Foxp3^+^ of each group of mice is represented. Isolated Cd4^+^/Cd25^+^ Treg were maintained in RLT Buffer with β-Mercaptoethanol and stored at -80°C until use.

### 2.4 DNA isolation and Whole genome bisulphite sequencing

DNA of Treg cells from spleen was extracted using DNeasy Blood & Tissue Kit (Qiagen, Hilden, Germany) according to manufacturer’s recommendations. DNA quality control, bisulphite conversion, library construction, and sequencing were performed by Novogene (Beijing, China) (26).

### 2.5 Bioinformatic analysis

To obtain the methylation ratio of each cytosine in CpG context, the following workflow was applied to the sequenced libraries. In first place, WGBS-seq libraries were processed by Trimmomatic (27), to trim low-quality and adapter base to ensure the quality of the samples. After that, trimmed fastq files were aligned with the bisulphite sequencing aligner bwa-meth (28) against mouse genome (GRCm39), and deduplicated with Picard (29). Finally, CpG context methylation was extracted with MethylDackel (30). Sample coverage and methylation statistics obtained after this procedure are detailed in **Table S1**.

After processing the raw data to obtain cytosines methylation values, differential methylation between groups was assessed. This analysis was performed with Methylikit (31), sliding window-based algorithm, which runs through the genome in windows of 1,000 bp and advancing 100 nucleotides with each step. Besides, each one of these windows was tested for differential methylation and then filtered according to the following criteria: regions with a *q*-value < 0.05 (adjusted *p*-value), and with at least 10 cytosines in the CpG context, were marked as differentially methylated, and those that did not overlap with gene promoters were filtered out. Thus, promoters were defined with a ChiPseeker annotatePeak function and covers 3 kb upstream and 3 kb downstream of the transcription starting site (32). Moreover, top 20% of the significant promoter regions were selected based on absolute methylation difference between groups (bigger than ± 7.5% methylation difference) to obtain differentially methylated promoter regions (DMPRs). Afterwards, DMPRs were annotated with ChiPseeker to their specific genes (32).

Finally, functional enrichment, gene ontology (GO), REACTOME and KEGG pathways of the genes affected by DMPRs were conducted with clusterProfiler R package (33). Additional figures and diagrams were done with VennDiagram (34), clusterProfiler (33), and EnhancedVolcano (35). All processing and analysis workflow were carried out according to previous work (22).

### 2.6 Statistical analyses

Data is expressed as mean ± standard deviation (SD). Normality was analyzed using the Shapiro-Wilk test. Paired groups were compared by Mann-Whitney test, and multiple groups were contrasted by Kruskal-Wallis test with Dunn’s *post hoc* test. Statistical software GraphPad Prism 8 (GraphPad Software Inc., San Diego, CA, USA) were used for statistical analysis.

## 3 Results

### 3.1 Induction of tolerance or desensitization by D1ManPrup3-SLIT

Anaphylactic or tolerant responses were confirmed one or five weeks after the final dose of SLIT by challenging the mice with Pru p 3. Results are the same as previously published (22), because we used the same mice to develop the present study, but with different samples from them, in this case spleen. Noteworthy, when the challenge was performed one week after only anaphylactic mice showed a significant decrease in body temperature after challenge (Basal: 38.08 ± 0.08°C vs Challenged: 35.00 ± 0.67°C, *p* < 0.001), whereas the rest of groups did not change their body temperatures after challenge and no further severe behavioral symptoms linked to anaphylactic reactions were observed (22). Likewise, serum IgE and IgG_1_ levels were significant lower in tolerant and desensitized mice in comparison to anaphylactic group: tolerant vs anaphylactic (sIgE: 0.056 ± 0.005 vs 0.101 ± 0.011 OD 450 nm, *p* < 0.01; and sIgG_1_: 0.251 ± 0.365 vs 2.563 ± 0.581 OD 450 nm, *p* < 0.05), and desensitized vs anaphylactic (sIgE: 0.059 ± 0.004 vs 0.101 ± 0.011 OD 450 nm, *p* < 0.05; and sIgG_1_: 0.451 ± 0.561 vs. 2.563 ± 0.581 OD 450 nm, p < 0.05) (22).

Tolerant and desensitized responses were also assessed five weeks after the last dose of D1ManPrup3-SLIT, and tolerance was observed in mice treated with 2 nM D1ManPrup3, but not in mice treated with 5 nM D1ManPrup3. Only desensitized mice suffered a significant drop in the body temperature (Basal: 38.07 ± 0.23°C vs Challenged: 36.76 ± 1.21°C, *p* < 0.05), whereas the temperature of tolerant group was similar before and after challenge (Basal: 38.14 ± 0.23°C vs Challenged: 37.45 ± 1.84°C, *p* > 0.05). Likewise, sIgE and sIgG_1_ levels were higher in desensitized mice in comparison to tolerant group (sIgE: 0.064 ± 0.007 vs. 0.063 ± 0.015 OD 450 nm; and sIgG_1_: 0.512 ± 0.428 vs. 0.102 ± 0.076 OD 450 nm). Therefore, these data confirm tolerant responses or temporary desensitization.

### 3.2 Treg purity and whole genome bisulphite sequencing and read alignment

After Treg isolation, the purity of Cd4^+^/Cd25^+^ cells was around 75%. Besides, a high percentage of Cd4^+^/Cd25^+^ cells were also Foxp3^+^ (80-99%), confirming that the analyzed cells were Treg (**Figure 1**). Although the remaining percentage of non-Treg cells or the different number of Treg cells found among groups could affect the analysis, it is important to highlight that these percentages of purification were similar in all groups and we did not find any significant differences (**Table 1**). This indicates that the possible cell impurity or the percentage of Treg obtained interfere in neither the analysis nor the differences found between groups.

**Table 1:** Percentage of Cd4^+^/Cd25^+^ and Foxp3^+^ cells obtained after Treg cells isolation.

GROUP	CELL POPULATION	PROPORTION (%)	STATISTICS
ANTIGEN-ONLY	Cd4^+^/Cd25^+^	75.43 ± 5.24	*p* > 0.05 (N.S.)
	Foxp3^+^	88.93 ± 8.04	
ANAPHYLAXIS	Cd4^+^/Cd25^+^	74.74 ± 7.92	
	Foxp3^+^	84.16 ± 11.73	
TOLERANT/2nM	Cd4^+^/Cd25^+^	78.19 ± 5.36	
	Foxp3^+^	82.74 ± 3.77	
DESENSITIZED/5nM	Cd4^+^/Cd25^+^	74.64 ± 5.54	
	Foxp3^+^	87.29 ± 6.34	

Comparisons were performed among all groups using the percentage of Cd4^+^/Cd25^+^ or Foxp3^+^ cells. Results are expressed as mean ± SD. N.S., not significant.

Thus, 26 high quality DNA samples from Tregs of mouse spleen, with a bisulphite conversion rate of > 99%, were sequenced to obtain 26 libraries containing approximately 300.000.000 reads per sample. Then, libraries were aligned and deduplicated resulting in around 80% of aligned reads after deduplication, which achieved an average of 20x mean coverage for each chromosome. Moreover, 80% of the cytosines covered in the CpG context were methylated as expected.

### 3.3 D1ManPrup3 SLIT shapes Treg methylome in a dose dependent manner

Antigen-only, tolerant, and desensitized groups of mice were compared against anaphylactic group to examine the methylation differences arising from SLIT treatment, which is the primary aim of our study. Groups were contrasted, and the hypermethylated/hypomethylated regions displayed higher/lower methylation in the samples belonging to the anaphylactic group. In addition, our study’s initial focus was differentially methylated regions overlapping with genes promoters, because methylation changes in these areas are more likely to be linked to changes in gene expression.

**Figure 2:**
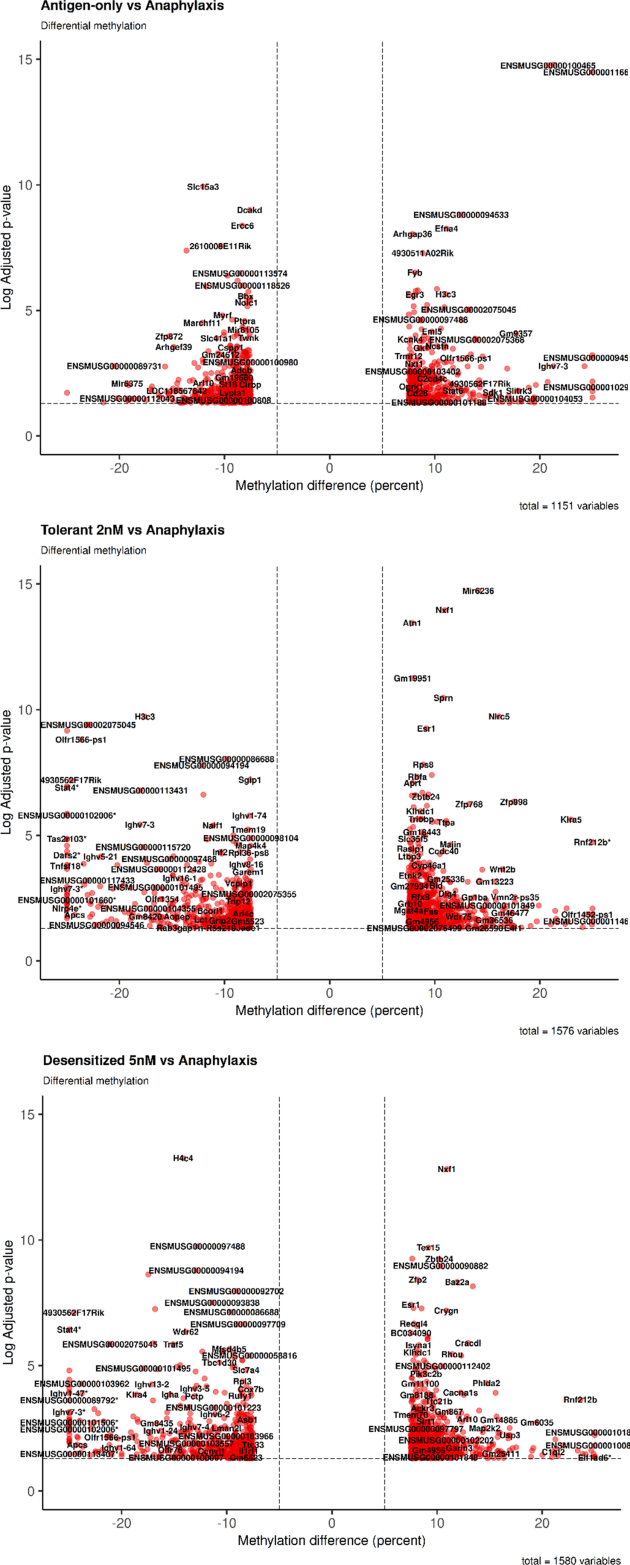
General changes observed in cytosines methylation of promoters of the genes. Volcano plot were performed for each comparison. *DMPR with abs (methylation difference) > 30%.

Firstly, as shown in **Table 2**, in both SLIT-treated group comparisons detected a roughly equal number of DMPRs, 1,576 DMPRs for tolerant and 1,580 DMPRs for desensitized, more than 400 DMPRs those found for antigen-only group. In addition, DMPR numbers of antigen-only comparison differed from the SLIT comparisons in the number of hypermethylated regions detected, which accounts for almost two thirds of SLITs DMPRs and only 40% of antigen-only DMPRs. On the other hand, the number of hypomethylated regions was similar in all three comparisons. Additionally, these data can be observed in the dispersion of DMPRs from the center of the volcano plots (**Figure 2**), corroborating the differences showed in **Table 2**, as hypermethylated changes in SLIT-treated groups were higher in magnitude and significance in comparison to antigen-only group. Full details of the DMPRs found are shown in the **Table S1**. Additionally, **Table S2** shows all differential methylated regions found in the analysis performed.

**Table 2:** Summary table of the differentially methylated promoter regions found in the study.

Differentially Methylated Regions	Antigen-only versus anaphylaxis	Tolerant/2nM versus anaphylaxis	Desensitized/5nM versus anaphylaxis
All	1151	1576	1580
Hypermethylated	469	951	953
Hypomethylated	682	625	627
Top 50 (by adjusted p-value)	*H4c14, Slc15a3, Dcakd, Ercc6, Efna4, Arhgap36, 2610008E11Rik, Dpy19l4, 4930511A02Rik, Fyb, Tex15, Six5, Ids, Eda, Nhs, Zfp703, H3c3, Egr3, Mdga1, Bbx, Plekhg5, Unc45a, Nolc1, Gm715, Dhrs4, Kcnc1, Dock11, n-R5s149, Carhsp1, Myrf, Ddx10, Aopep, Bcl3, Ptpra, Marchf11, Ccdc149, Fam120c, 2310030G06Rik, Mir8105, Dhh, Eml5, Plk3, Azin2, Gm9357, Etfdh, Proser3, Zfp277, Irak1, Tent5d, Zfp872*	*H2ac20, Trim17, H3c6, Pik3r6, Mfsd6l, H3c7, Rap1b, H4f16, H4c4, Mir6236, Nxf1, Atn1, Gm19951, Sprn, Nlrc5, H3c3, Esr1, Olfr1566-ps1, Rps8, Rbfa, Sgip1, 4930562F17Rik, Aprt, Stat4, Tmeff1, Lama1, Gm3945, Megf8, Rprd1a, Zbtb24, Zfp998, Zfp768, Dag1, 4930520O04Rik, Klhdc1, Ighv1-74, Gm960, Triobp, Mir6956, Klra5, Svep1, Sun1, Tmem223, Pde1b, Ttpa, Ighv7-3, Slc25a25, Naif1, Man1c1, Mir1983*	*Bola1, H2ac21, H2ac20, Trim17, H3c6, Pik3r6, Mfsd6l, H3c7, H4c4, Nxf1, Tex15, Arhgef33, Zbtb24, H3c3, Zfp2, Baz2a, Nlrc5, Esr1, Gnas, Baalc, Crygn, 4930562F17Rik, Recql4, Marveld2, Stat4, Wdr62, BC034090, Samhd1, Rom1, Eml3, Cracdl, Adam33, Traf5, Isyna1, Mfsd4b5, Gys1, Slc25a25, Naif1, Clec2g, Klhdc1, Rhou, Kdm2b, n-R5s149, Eci3, Chchd7, Mzf1, Tbc1d30, Tcf7l1, Lin52, Rusc2*
Number of genes affected	1126	1529	1536

Adjusted p-value < 0.05 and abs (methylation difference) >=5%.

**Figure 3:**
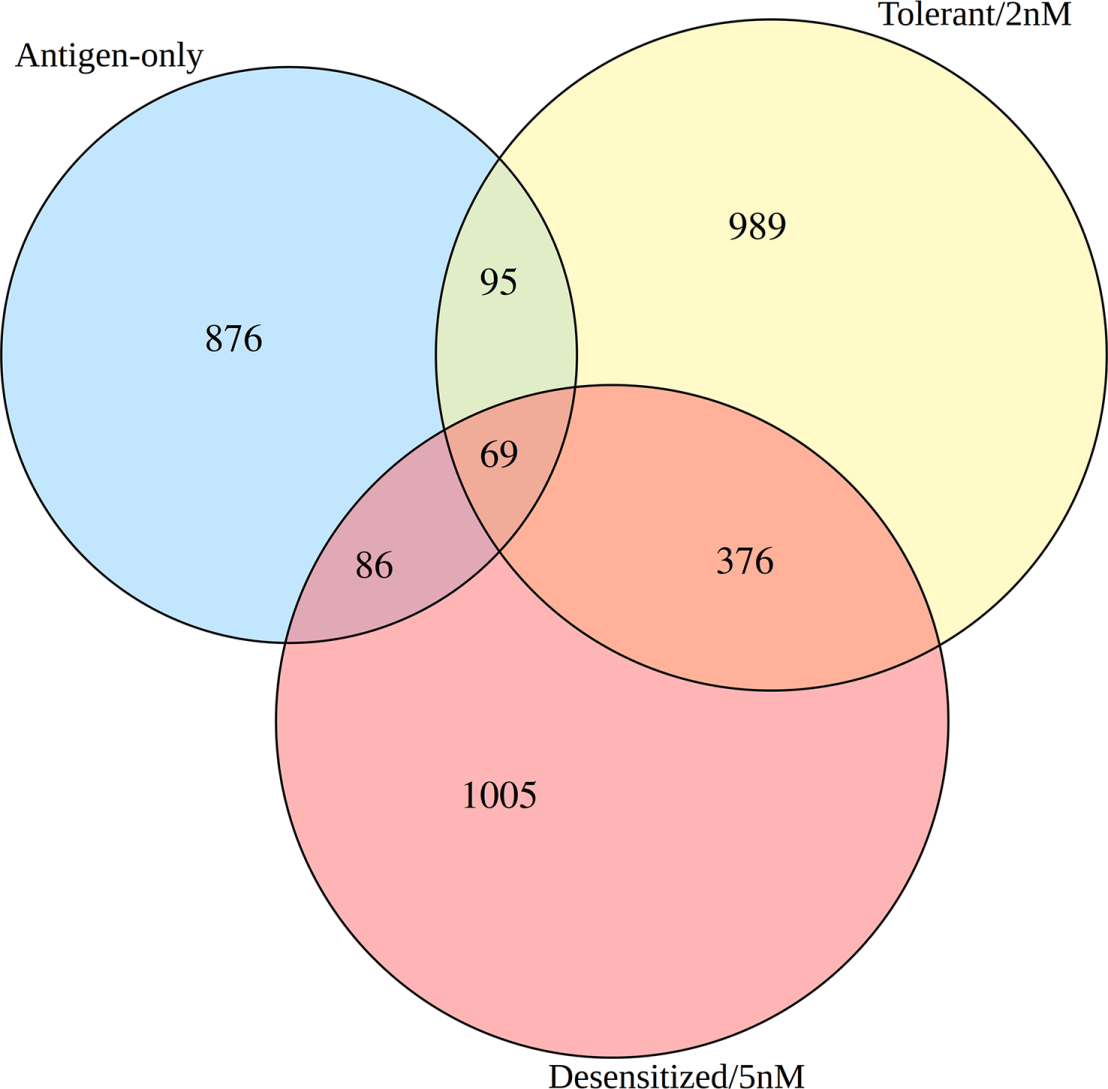
Venn diagram of the differentially methylated promoter regions found in the three comparisons.

Secondly, although differential methylation in SLIT comparisons seemed to be quantitative similar, SLIT dosage induced two distinctive phenotypes after a few weeks and Tregs are expected to have a leading role, so we needed to explore the differences between them. To compare DMPR lists with each other at identity level, a three-way Venns diagram was done (**Figure 3** and **Table S1**). Only 69 genes were affected by common DMPRs found in the three groups, and tolerant and desensitized mice shared more DMPRs between them than with antigen-only group (**Figure 3**). Moreover, tolerant and desensitized groups only shared 445 genes with at least one DMPR, despite them having similar number of DMPRs. Therefore, we found several DMPRs among the different groups, although most of them did not overlap. These overall results showed that the SLIT with D1ManPrup3 affects Tregs, differentiating them from the Tregs of the untreated animals: anaphylactic and sensitized. Moreover, another outcome found was the tolerant responses induced by SLIT, although clinically similar, were not completely comparable with natural tolerance, as expected.

Furthermore, D1ManPrup3 dose induces distinctive methylation changes that are potentially related to long-term phenotypic differences linked to Pru p 3 tolerance.

### 3.4 SLIT treatment leads to differential methylation in genes associated with Tregs immunomodulation in a dose-dependent manner

To study the biological meaning and to explore the processes in which the DMPRs observed could be engaging in, several functional enrichments in terms of GO, REACTOME and KEGG were performed. In **Table S3**, GO functional enrichment with the category of biological processes is summarized. Antigen-only enrichment consisted in a series of terms related to lymphocytes T cellular biology, including activation (positive regulation of lymphocyte activation), differentiation (regulation of lymphocyte differentiation), and interaction with other cells (regulation of cell-cell adhesion). Moreover, both tolerant and desensitized enrichments consisted in a core of terms linked to a lymphocyte, specifically B cell, mediated immune response, such as B cell activation, cell recognition, lymphocyte mediated immune response, and defense response to bacterium, and various not immune related pathways that could be enriched by DMPRs affecting ubiquitous genes (**Table S3**). Unsurprisingly, GO functional enrichment showed that the DMPRs were involved in cellular and systemic processes concerning the non-anaphylactic response to Pru p 3. Additionally, KEGG and REACTOME functional enrichments were performed; however, no relevant results were obtained (**Table S1**).

**Figure 4:**
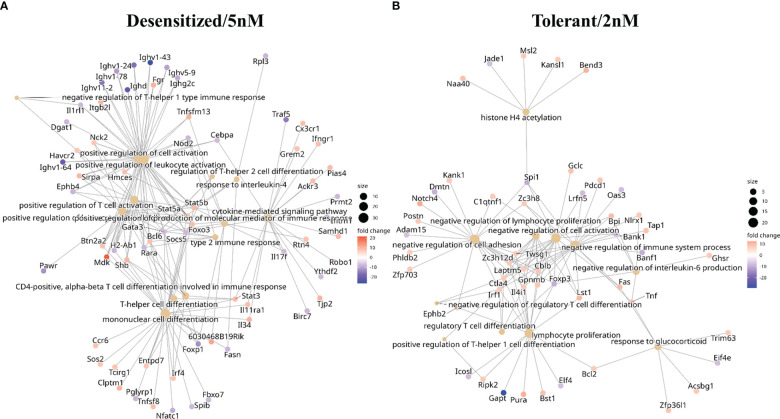
Functional enrichment in biological processes GO terms obtained with genes affected by at least one differentially methylated promoter regions only in one SLIT comparison. **(A)** Tolerant/2nM vs anaphylaxis comparison. **(B)** Desensitized/5nM vs anaphylaxis comparison.

As shown in **Figure 3**, tolerant and desensitized groups only shared 445 genes, had 989 and 1005 exclusive genes respectively, and still obtained a similar global enrichment. To explore which biological functions were overrepresented in the gene sets exclusive of each SLIT group, an additional functional enrichment was conducted (**Figure 4** and **Table S1**). The enrichment of the set of genes affected by DMPRs in both SLIT comparisons confirmed that the global enrichment shown before was due to this set (**Table S1**). In addition, in 13 of these 445 genes, although each SLIT comparison detected a DMPR in its promoter, the changes had opposite directions (**Table S1**). These 13 genes obtained enriched terms like lymphocyte differentiation, positive regulation of type 2 immune response and isotype switching to IgE isotypes (**Table S1**).

Back to the point, in **Figure 4** a collection of highlighted immune related enriched terms, and the genes enriching them, are depicted. In desensitized mice exclusive affected genes enrichment (**Figure 4A**) terms like regulation of T-helper 2 cell differentiation or type 2 immune response were found as overrepresented by genes such as *Stat3*, *Stat5a*, *Stat5b*, *Gata3*, *Foxo3*, *Il34*, and *Irf4*. Conversely, for tolerant exclusive affected genes terms such as negative regulation of regulatory T cell differentiation, response to glucocorticoid and negative regulation of interleukin-6 production were enriched by DMPRs in genes like *Tnf*, *Irf1*, *Foxp3*, and *Ctla4* (**Figure 4B**). This showed that SLIT dosage induced divergent regulation pathways that could be responsible for long-term differences found in tolerant and desensitized mice.

### 3.5 Different D1ManPrup3 dose affects different Treg cells key transcription factors

Finally, as noted in the previous section, many transcription factors were related to biological processes regulated by the methylation changes detected against the anaphylactic group. These transcription factors may have been involved in the cellular programming that governs the long-term tolerogenic behavior of regulatory T cells. Therefore, the lists of DMPRs obtained above were filtered to highlight affected genes that are annotated as transcription factors or co-factors (**Table 3**). Moreover, **Table 4** shows the genes affected in both comparisons for the SLIT treated groups, sorted whether they are affected in both or only in one of them. Some of the most notable are *Foxp3*, *Irf1*, *Relb*, *Irf8*, and *Myo6* in tolerant/2nM exclusives, and *Foxp1*, *Gata3*, *Stat5a*, and *Stat5b* in desensitized/5nM.

**Table 3:** Transcription factors and co-factors affected by DMPRs in each comparison.

Differentially Methylated Regions	Antigen-only versus anaphylaxis	Tolerant/2nM versus anaphylaxis	Desensitized/5nM versus anaphylaxis
All	98	106	109
Hypermethylated	33	75	70
Hypomethylated	65	31	39
Top 50 (by adjusted p-value)	*2610008E11Rik, Six5, Zfp703, Egr3, Bbx, Carhsp1, Myrf, Bcl3, Plk3, Zfp277, Zfp872, Prox2, Cdk5, Ciz1, Tfe3, Zfp599, Snx6, Zscan22, Esx1, Pbx2, Tcf20, Fhl1, Tcf7, Tsc22d3, Mbtps2, Zfp874a, Hmgb3, Mbd2, Arx, Pdcd4, Ash2l, Spz1, Hic2, Foxo3, Zfp982, Zfp998, Stat5b, Alyref, Rorc, Zbtb3, Mycbp2, Rfx5, Foxn3, Tfe3, Foxp3, St18, Snw1, Rbpjl, Gata6, Cd3d*	*Esr1, Stat4, Rprd1a, Zbtb24, Zfp998, Zfp768, Tonsl, Gm14295, Vsx1, Tcf4, Zbtb33, Mzf1, Arx, Elf4, Tsc22d1, Prdm6, Hdac2, Zfp36l1, Med27, Zfp703, Rfx8, Zfp972, Relb, Snx6, Trip4, Kank1, Fbp1, Zfp111, Zfp982, Tead2, Zfp958, Tshz1, Zfp951, Pcbd1, Zfp568, Hdac7, Esrrb, Eya2, Bend3, Prdm12, Gtf2a1l, Zfp316, Bhlha15, Gata6, Helz2, Jdp2, Nr1h2, Zfp277, Zfp512, Cbx6*	*Zbtb24, Zfp2, Baz2a, Esr1, Stat4, Kdm2b, Mzf1, Tcf7l1, Zbtb41, Zfp748, Gata6, Snapc2, Arx, Ybx3, Tonsl, Foxg1, Calcoco1, Zfp382, Bcl6, Chd3, Prmt2, Rfx8, Zfp972, Gm14295, Zfp583, Pdlim1, Bcl9, Zfp59, Sirt1, Grip1, Irf6, Fbp1, Zfp473, Flna, Cdc73, Mdm4, Actn1, Tead2, Zfp316, Foxo3, Gm9049, Irf4, Egln1, Nfatc1, Mzf1, Sp7, Ncoa3, Zmynd8, Mbd2, Nfe2*
Number of Transcription factors and cofactors affected	96	105	106

Adjusted p-value < 0.05 and abs (methylation difference) >=5%.

**Table 4:** DMPRs on interesting genes observed in tolerant/2nM and desensitized/5nM comparison.

Transcription factors DMPRs	Tolerant/2nM AND desensitized/5nM	Desensitized/5nM	Tolerant/2nM
Number	28	78	77
Symbol	*Rfx8,Stat4,Mdm4,Esr1,Zbtb24,Sirt1,Tnip1,Esrrb,Fbp1,Zfp998,Elp3,Zfp622,Tonsl,Sp7,Gata6,Rprd1a,Tshz1,Ankrd1,Gm14295,Zfp972,Zfp268,Kdm2b,Zfp316,Mzf1,Tead2,Zfp768,Flna,Arx*	*Npas2,Klf7,Zbtb41,Cdc73,Esrrg,Irf6,Olig3,Bclaf1,Foxo3,Prmt2,Pias4,Gm10778,Pawr,Gm9049,Grip1,Baz2a,Zfp2,Trim16,Chd3,Rara,Stat5b,Stat5a,Stat3,Foxg1,Actn1,Foxn3,Pou6f2,Irf4,Zfp748,Rarb,Grhl2,Zfp623,Nr4a1,Calcoco1,Nfe2,Bcl6,Nrip1,Zfp995,Zfp943,Pou5f1,Epc1,Mbd2,Nfatc1,Pdlim1,Gata3,Zfp341,Phf20,Zmynd8,Ncoa3,Myt1,Bcl9,Bach2,Zfp618,Foxe3,ENSMUSG00000086147,Ctbp1,Jazf1,Tcf7l1,Foxp1,Ybx3,Zfp583,Crxos,Zfp59,Zfp566,Zfp382,Cebpa,Spib,Zfp473,Phox2a,Arntl,Snapc2,Nod2,Egln1,Rnf111,Zfp275,Mecp2,Zxdb,Trappc2*	*Kdm5b,Zfp281,Nr5a2,Hdac2,Bend3,Pcbd1,Tfam,Nr2c1,Irf1,Phf12,Tbx4,Vezf1,Med1,Alyref,Zfp277,Snx6,Baz1a,Six1,Zfp36l1,Jdp2,Actn2,Gm5141,Zfp712,Hnrnpc,Nfatc4,Tsc22d1,Rad21,Cbx6,Hdac7,Gtf2e1,E4f1,Notch4,Gtf2a1l,Pura,Prdm6,Tcf4,Foxd4,Kank1,Ldb1,Suv39h2,Med27,Prdm12,Spi1,Kcnip3,Vsx1,Rbpjl,Eya2,Helz2,Jade1,Alx3,Arid3c,Zfp991,Zfp982,Rere,Trp73,Zfp512,Zfp951,Bhlha15,Hdac11,Relb,Zfp111,Zfp60,Zfp568,Zfp975,Nr1h2,Klf13,Zfp958,Zfp703,Dusp26,Irf8,Pml,Trip4,Myo6,Foxp3,Zbtb33,Elf4,Fhl1*

Transcription factors of interest are highlighted in blue (hypomethylated) and in red (hypermethylated).

## 4 Discussion

In this work, we provided evident differential methylation pattern in splenic-Treg cells from an anaphylactic mouse model treated or not with a synthetic glycodendropeptide (D1ManPrup3). Several types of mechanisms are involved in the regulation of gene expression, but cytosine methylation is one of the key elements in gene regulation (36). Moreover, these methylation profiles are different according to the administered dose and their effects, which could have an important impact on Treg functions. In addition, it could indirectly affect key Treg genes involved in tolerance acquisition after immunotherapy. As Treg cells are linked to tolerance mechanisms, the methylation changes found in this cell type could explain differences in the desensitization or tolerant events observed in mice after D1ManPrup3-SLIT at different doses, and subsequently, the principal mechanisms in long-lasting tolerance.

As we expected, and in line with our previous results, the dose of D1ManPrup3 administered in SLIT induces tolerance or desensitization, and indirectly changes in the methylation profile in a key cell type for tolerance acquisition: Treg cells (22). This fact offers the possibility of studying the mechanisms that govern tolerance at molecular level and, additionally, it could provide an important knowledge in to discern methylation changes that occur during desensitization or tolerance at molecular level. There is more evidence that the mechanisms that induce tolerance or desensitization are different at molecular level and depending on key cell types for tolerance acquisition (22, 37).

The most relevant DMPRs found in this study are those involved in Treg tolerance enhancement. In fact, multiple transcription factors that govern Tregs behavior and differentiation were affected by at least one DMPR in one of the two SLIT comparisons. Indeed, *Stat4* was found as differentially methylated in both treated-mouse groups and Stat4 ratio under Foxp3 defines effector Tregs by preventing Tregs identity destabilization by inflammatory conditions (38); in addition, *Stat4* expression is controlled by its promoter methylation (39). In this study we found one hypomethylated DMPR in each comparison, which, if a canonical relationship is assumed, should increase *Stat4* expression. Moreover, we found more hypomethylation in the temporally desensitized mice group. However, although *Stat4* is potentially more expressed in both SLIT receiving mice group, *Foxp3* is also affected by a hypomethylated DMPR in tolerant animals, potentially even increasing Foxp3/Stat4 ratio. In the same way, other effector Treg signature ratio described in Cuadrado et al., Foxp3/Foxp1 in desensitized/5nM group a hypomethylated DMPR was found in *Foxp1* promoter, which could possibly result in a lower Foxp3/Foxp1 ratio typical of non-effector Tregs (38). These differential methylation marks that differentiate tolerance from desensitization could be linked to the ability of Tregs to develop their effector capacity under inflammatory conditions.

What is more, *Foxp3* regulatory elements methylation controls its expression (40) and, therefore, also the influence of Tregs in tolerance on dietary antigens (41). *Foxp3* promoter decreased methylation found in tolerant mice seemed to point to the same direction. Supporting these findings, in previous works applying identical SLIT with D1ManPrup3 in a similar Pru p 3-induced anaphylaxis mouse model, higher levels of Foxp3 were observed in the tolerant group compared to desensitized animals, which also translated into higher IL-10 levels (14). All these events make *Foxp3* DMPR a promising biomarker of effective immunotherapy. Nevertheless, additional factors such as alternative splicing (42) or co-expressed regulators should be considered (43). These data are in concordance with previous results, where an hypomethylation of *Foxp3* was observed in an experimental mouse model to peanut treated after epicutaneous immunotherapy explaining the sustainability of protection (44), which could be involved in the efficacy of immunotherapy.

Besides, our results show that *Gata3* was only hypomethylated in the desensitized mice and not differentially methylated in tolerant group. This transcription factor is considered as the “master regulator” for Th2 cells (45), having pro-inflammatory effects and being essential for the development and proliferation of Th2 cells (46). The hypomethylation found in this transcription factor could be indicative of loss of effectiveness of SLIT in desensitized group. Nevertheless, it is important to consider that stability between Foxp3 and Gata3 is required to maintain homeostasis and a stable Treg phenotype.

Another important finding that could be linked to immunotherapy efficacy responses is the altered methylation profile in the *Stat5a* and *Stat5b* transcription factors, which was only found in desensitized mouse. STAT5 is essential for the development and maintenance of Treg cells (47), because it can inhibit the production of IL-17 and prevent the differentiation of Tregs into Th17 cellular phenotype (48). Moreover, STAT5 plays a key role in IgE-mediated cytokine production and release of histamine and leukotriene (49). Moreover, it should be taken into account that these transcription factors, especially *Stat5b*, are directly involved in the correct expression of *Foxp3*, so that an alteration in their expression could also affect *Foxp3* gene expression and, subsequently, susceptibility to allergic disease (50). In this sense, and the same line with the results found in our study, the fact that *Stat5b* was found differentially methylated in the desensitized group may eventually cause the abnormal expression of FoxP3 and affect the tolerance mechanisms triggered by Treg.

Finally, it is important to highlight that these results have been obtained from splenic-Treg of mice, just at the end of the SLIT, at the same time-point that no anaphylactic symptoms were observed after Pru p 3 challenge in any treated group: tolerant or desensitized. This fact could mean that although no clinical and immunologically differences were observed between treated groups, epigenetic differences found at this point could reflect a different immunological response in the period of time after stopping the treatment. These are important findings suggesting that these biomarkers could be used to predict a better long-term response, either tolerance or desensitization.

Despite these promising results, we are aware of the limitations of our study, which would lead to the need for corroborating these results, especially at the gene and protein expression level. Furthermore, it is a proof-of-concept screening of methylation changes focused on promoter regions from Treg genome, missing other genomic regions, which could be potentially interesting. Despite this, changes in methylation of promoter regions are good long-term biomarkers, as these epigenetic changes persist over time and are even passed on to offspring (51). However, there is not always a canonical relationship between methylation and gene expression (52); thus, these changes have to be studied at the genetic and protein level and in relation to the mechanisms in which they participate. Nevertheless, these epigenetic changes found in Treg cells are expected to be long-lasting and transferable to humans, which could be used as promising biomarkers of tolerance response and, subsequently, to the efficacy of immunotherapy. Although these biomarkers have to be taken with caution, and need to be confirmed due to the Treg purification ratio of the isolation kit used. Therefore, furthers studies are needed.

In conclusion, this study reports diverse methylation changes in key genes and transcription factors in Treg cells from spleen of mouse with different dosage of D1ManPrup3 in SLIT, which could be involved in the acquisition of long-term food tolerance. Most DMPRs found in this study were exclusive to each group of mice, although some of them were common to two groups or among all groups of mice studied (antigen-only, tolerant and desensitized). Remarkably, we found that *Foxp3* appeared exclusively hypomethylated in tolerant mice, whereas *Gata3* was only hypomethylated in the desensitized group, which could mean that these transcription factors can influence tolerance or desensitization by inducing several immunological responses, since these transcription factors play an important role in pro-inflammatory or regulatory responses in Treg cells, and specifically, in the long-term tolerant responses against Pru p 3. Thus, these methylation marks and their affected genes from Treg cells could be used as potential biomarkers to differentiate a successful and effective immunotherapy effects or a temporary desensitization.

## Data availability statement

The data presented in the study are deposited in the Bioproject repository, accession number PRJNA937049.

## Ethics statement

The animal study was reviewed and approved by Animal Experimentation Ethics Committee of Andalusian Centre for Nanomedicine and Biotechnology (BIONAND, Malaga, Spain).

## Author contributions

Conceptualization, MT, JC and CM. Methodology, RN, MR, CL-M, MM-A, JR-S, JR and JC. Formal analysis, RN, MR, CL-M and JC. Data curation, RN, MR, CL-M, JC and CM. Original draft preparation, RN, CL-M, MM-A, JC and CM. Review and editing, MT, JC and CM. Supervision, JR, MT, JC and CM. Funding acquisition, MT, JR and CM. All authors contributed to the article and approved the submitted version.
