# Supplementary material for: A synthetic glycodendropeptide induces methylation changes on regulatory T cells linked to tolerant responses in anaphylactic-mice
Source: Front Immunol. 2023 Jun 2;14:1165852. doi: 10.3389/fimmu.2023.1165852 (PMC10272618; doi:10.3389/fimmu.2023.1165852)
Supplement: Supplementary file 1 [file DataSheet_1.docx]

Supplementary Material

A synthetic glycodendropeptide induces methylation changes on regulatory T cells linked to tolerant responses in anaphylactic-mice

Rafael Núñez^1†^, María J. Rodríguez^1†^, Clara Lebrón-Martín^1†^, María del Carmen Martín-Astorga^1,2^, Javier Ramos-Soriano^3^, Javier Rojo^3^, María J. Torres^1,2,4‡^, José Antonio Cañas^1*‡^, Cristobalina Mayorga^1,4*‡^

^1^Laboratory of Allergy, Allergy Research Group, Instituto de Investigación Biomédica de Málaga-Plataforma BIONAND (IBIMA-BIONAND), Málaga, Spain

^2^Department of Medicine, Universidad de Málaga (UMA), Málaga, Spain

^3^Laboratory of Glycosystems, Institute of Chemical Research (IIQ), CSIC - Universidad de Sevilla, Sevilla, Spain

^4^Clinical Unit of Allergy, Hospital Regional Universitario de Málaga, Málaga, Spain

† These authors contributed equally to this work and share first authorship

‡ These authors contributed equally to this work and share last authorship

***Correspondence:**Cristobalina Mayorga
lina.mayorga@ibima.eu

José Antonio Cañas

joseantonio.canas@ibima.eu

# Supplementary Data

Table S1 shows methylation statistics, coordinates of DMPRs found and GO, KEGG and REACTOME comparisons.

Table S2 shows all differential methylated windows found in the analysis performed.

# Supplementary Tables

**Table S3.** Biological processes GO terms enriched for each comparison. All enrichments were performed by comparison with anaphylactic mice.

| **ANTIGEN-ONLY vs ANAPHYLACTIC** | | | **TOLERANT vs ANAPHYLACTIC** | | | **DESENSITIZED vs ANAPHYLACTIC** | | |
| --- | --- | --- | --- | --- | --- | --- | --- | --- |
| **ID** | **Description** | **Adjusted *p*-value** | **ID** | **Description** | **Adjusted *p*-value** | **ID** | **Description** | **Adjusted *p*-value** |
| GO:0030098 | Lymphocyte differentiation | 0.00 | GO:0050853 | B cell receptor signaling pathway | 0.00 | GO:0002455 | Humoral immune response mediated by circulating immunoglobulin | 0.00 |
| GO:0045619 | Regulation of lymphocyte differentiation | 0.00 | GO:0050864 | Regulation of B cell activation | 0.00 | GO:0006958 | Complement activation, classical pathway | 0.00 |
| GO:0051251 | Positive regulation of lymphocyte activation | 0.00 | GO:0006910 | Phagocytosis, recognition | 0.00 | GO:0006910 | Phagocytosis, recognition | 0.00 |
| GO:0035710 | CD4-positive, alpha-beta T cell activation | 0.00 | GO:0006958 | Complement activation, classical pathway | 0.00 | GO:0050871 | Positive regulation of B cell activation | 0.00 |
| GO:0050851 | Antigen receptor-mediated signaling pathway | 0.00 | GO:0042113 | B cell activation | 0.00 | GO:0050853 | B cell receptor signaling pathway | 0.00 |
| GO:0046631 | Alpha-beta T cell activation | 0.00 | GO:0006911 | Phagocytosis, engulfment | 0.00 | GO:0008037 | Cell recognition | 0.00 |
| GO:0022407 | Regulation of cell-cell adhesion | 0.00 | GO:0099024 | Plasma membrane invagination | 0.00 | GO:0006911 | Phagocytosis, engulfment | 0.00 |
| GO:0002287 | Alpha-beta T cell activation involved in immune response | 0.01 | GO:0008037 | Cell recognition | 0.00 | GO:0099024 | Plasma membrane invagination | 0.00 |
| GO:0032753 | Positive regulation of interleukin-4 production | 0.01 | GO:0002460 | Adaptive immune response based on somatic recombination of immune receptors built from immunoglobulin superfamily domains | 0.00 | GO:0002460 | Adaptive immune response based on somatic recombination of immune receptors built from immunoglobulin superfamily domains | 0.00 |
| GO:0001764 | Neuron migration | 0.01 | GO:0042742 | Defense response to bacterium | 0.00 | GO:0042113 | B cell activation | 0.00 |
| GO:0050863 | Regulation of T cell activation | 0.02 | GO:0002449 | Lymphocyte mediated immunity | 0.00 | GO:0002449 | Lymphocyte mediated immunity | 0.00 |
| GO:0042113 | B cell activation | 0.02 | GO:0036159 | Inner dynein arm assembly | 0.01 | GO:0006959 | Humoral immune response | 0.00 |
| GO:1904861 | Excitatory synapse assembly | 0.02 | GO:0006959 | Humoral immune response | 0.01 | GO:0042742 | Defense response to bacterium | 0.00 |
| GO:0050870 | Positive regulation of T cell activation | 0.04 | GO:0099072 | regulation of postsynaptic membrane Neurotransmitter receptor levels | 0.01 | GO:0002285 | Lymphocyte activation involved in immune response | 0.00 |
| GO:0007159 | Leukocyte cell-cell adhesion | 0.04 | GO:0050867 | Positive regulation of cell activation | 0.03 | GO:0007015 | Actin filament organization | 0.00 |
|  |  |  | GO:0007015 | Actin filament organization | 0.03 | GO:1903131 | Mononuclear cell differentiation | 0.01 |
|  |  |  | GO:0050866 | Negative regulation of cell activation | 0.04 | GO:0042093 | T-helper cell differentiation | 0.01 |
|  |  |  |  |  |  | GO:0090132 | Epithelium migration | 0.01 |
|  |  |  |  |  |  | GO:0048608 | Reproductive structure development | 0.02 |
|  |  |  |  |  |  | GO:0010631 | Epithelial cell migration | 0.02 |
|  |  |  |  |  |  | GO:0044728 | DNA methylation or demethylation | 0.02 |
|  |  |  |  |  |  | GO:0061000 | Negative regulation of dendritic spine development | 0.04 |
|  |  |  |  |  |  | GO:0110053 | Regulation of actin filament organization | 0.04 |
|  |  |  |  |  |  | GO:0032271 | Regulation of protein polymerization | 0.04 |
|  |  |  |  |  |  | GO:1902903 | Regulation of supramolecular fiber organization | 0.05 |
|  |  |  |  |  |  | GO:0001892 | Embryonic placenta development | 0.05 |
